# The Importance of Transaminases Flare in Liver Elastography: Characterization of the Probability of Liver Fibrosis Overestimation by Hepatitis C Virus-Induced Cytolysis

**DOI:** 10.3390/microorganisms8030348

**Published:** 2020-02-29

**Authors:** Mauro Giuffrè, Sofia Fouraki, Manola Comar, Flora Masutti, Lory Saveria Crocè

**Affiliations:** 1 Department of Medical, Surgical and Health Sciences, University of Trieste, Trieste, 34149, Italy; drfouraki@gmail.com (S.F.); manola.comar@burlo.trieste.it (M.C.); lcroce@units.it (L.S.C.); 2 Italian Liver Foundation, Basovizza (Trieste) 34149, Italy; 3 Institute for Maternal and Child Health-IRCCS “Burlo Garofolo”, Trieste 34137, Italy; 4 Liver Clinic, Azienda Sanitaria Universitaria Giuliano-Isontina, Cattinara Hospital, Trieste 34149, Italy; fmasutti@units.it

**Keywords:** liver elastography, liver fibrosis, serum transaminase, HCV infection, ALT

## Abstract

Background: Liver stiffness measurement (LSM) is crucial for appropriate fibrosis staging in patients with ongoing hepatitis C virus (HCV) infection. However, there is still an ongoing debate on the impact of serum transaminases (aspartate-aminotransferase, AST; alanine-aminotransferase, ALT) on LSM. Methods: We selected 110 patients undergoing HCV eradication therapy with LSM compatible with significant liver fibrosis. LSM was evaluated prior to therapy and one year after HCV eradication. Results: LSM showed a median decrease of 35% from baseline values, and 67 (61%) patients showed posttreatment values compatible with lower fibrosis stages. We developed two logistic regression models to determine the probability of liver fibrosis overestimation according to serum transaminase. The probability of overestimation of two or more fibrosis grade is equal to (1) 50% for AST of 99 IU/L (2.2 ULN) and ALT of 90.5 IU/L (2 ULN), (2) 80% for AST of 123.5 IU/L (2.74 ULN) and ALT of 101.5 IU/L (2.25 ULN), and (3) reaches 100% for AST of 211 IU/L (4.7 ULN) and ALT of 140 IU/L (3.1 ULN). Conclusions: This study highlights the impact of serum transaminases on LSM. We believe that our findings may serve as a reference point for appropriate fibrosis stratification by liver elastography in patients with HCV infection.

## 1. Introduction

Hepatitis C Virus (HCV) infection represents a significant cause of chronic liver disease, with approximately 70 million chronically infected individuals worldwide [1]. Due to the lack of spontaneous virus clearance, most of the patients (approximately 55%–85%) develop chronic HCV infection, which over a period of 20–30 years could lead to liver cirrhosis in 10%–40% of cases [2]. Fortunately, the advent of second-generation direct antiviral agents (DAAs) has changed the natural history of chronic HCV infection [3]. Assessment of liver disease progression (i.e., liver fibrosis staging) is of particular importance, as the choice of the treatment regimen, post-treatment prognosis, and follow-up depend on the stage of fibrosis [4]. According to European Guidelines, noninvasive techniques (NITs) should be preferred to liver biopsy to assess liver fibrosis before therapy initiation [4]. Among the different NITS, liver elastography has revolutionized—due to its safety and accuracy—the everyday clinical quantification of liver fibrosis [5]. However, elastography is burdened by several confounding factors (such as serum transaminase flares, cholestasis, hepatic venous congestion, right heart failure, and steatosis) that could lead to inaccurate staging of liver fibrosis [6]. In particular, preliminary studies reported that virus-induced necroinflammatory activity, in the form of alanine transaminases (ALT) flares with values greater than ten times the upper limit of normal (ULN), may lead to falsely increased liver stiffness measurement (LSM) [7]. More recent findings showed that even patients with lesser ALT increase (≥2 ULN) had higher LSM if compared to patients with normal ALT [8,9]. In addition, this hypothesis was further explored through a significant decrease of LSM in patients with hepatitis B virus (HBV) infection after 3-months of antiviral treatment and ALT normalization [10]. Similar findings were found in a cohort of patients with HCV infection, in which patients with a higher histological degree of inflammation had higher LSM [11]. To definitively assess the influence of liver inflammation on liver elastography, we evaluated changes in LSM in a cohort of patients with HCV infection and LSM compatible with severe liver fibrosis one year after the end of treatment.

## 2. Material and Methods

The study was carried out following the guidelines of the local Ethics Committee for conducting research involving humans (ID: 2783—approved the 21 of May 2019). The study was conducted according to the criteria set by the declaration of Helsinki. Each subject signed informed consent before participating in the study.

All patients underwent HCV treatment evaluation and follow-up at the Liver Clinic (Ospedale Cattinara, Azienda Sanitaria Universitaria Giuliano Isontina) between January 2016 and February 2018 [12]. Prior to treatment (with a maximum of 14 days interval), each patient completed baseline laboratory testing including: HCV-genotype (determined with Gen-C 2.0), HCV-RNA quantification (normal range <15 IU/mL; measured with COBAS^®^ AmpliPrep/COBAS^®^ TaqMan^®^ HCV v2.0—Roche Molecular System, Inc., South Branchburg, NJ, USA), creatinine (normal range 0.5–1.3 mg/dL), glucose (normal range 65–110 mg/dL), total cholesterol (normal range <200 mg/dL), aspartate-aminotransferase (AST; normal range <45 IU/L), alanine-aminotransferase (ALT; normal range <45 IU/L), gamma-glutamyl transpeptidase (GGT; normal range <10–55 IU/L), alkaline phosphatase (ALP; normal range 30–120 IU/L), total bilirubin (normal range 0.3–1.2 mg/dL), albumin (normal range 3.5–5.2 d/dL), platelet count (normal range 150–450 × 10^3^/µL), and international normalized ratio (INR; normal range 0.8–1.1; measured with HemosIL Recombiplastin 2G). We also measured their body weight and height and calculated their Body Mass Index (BMI). 

Treatment regimens were selected according to the Agenzia Italiana del Farmaco (AIFA) criteria, that considered HCV genotype, diagnosis of liver cirrhosis, previous HCV treatment failure, chronic kidney disease, concurrent hepatocellular carcinoma or listing for liver organ transplantation. Accordingly: (1) 12 patients were treated with an eight week regimen of glecaprevir/pibrentasvir, (2) 85 patients with a twelve week regimen of sofosbuvir/simeprevir (*n* = 3), sofosbuvir/ribavirin (*n* = 9), sofosbuvir/daclatasvir (*n* = 9), sofosbuvir/velpatasvir (*n* = 30), glecaprevir/pibrentasvir (*n* = 16) ombitasvir/paritaprevir/ritonavir/dasabuvir (*n* = 15), elbasvir/grazoprevir (*n* = 3); (3) 9 patients with a sixteen week regimen of sofosbuvir/ribavirin (*n* = 3) and elbasvir/grazoprevir/ribavirin (*n* = 6), and (4) 4 patients with a twenty-four week regimen of sofosbuvir/ribavirin.

The main endpoint of therapy was defined as a sustained viral response (SVR), equivalent to undetectable HCV-RNA (≤15 IU/mL) in serum or plasma 12 weeks (SVR12) or 24 weeks (SVR24) after the end of therapy [13]. Laboratory tests were repeated every four weeks during the treatment and three months after the end of therapy and at the one-year interval after SVR12.

### 2.1. Ultrasound and Elastography

Both ultrasound and elastography examinations were performed using Philips Affiniti 70 (Philips Healthcare, The Netherlands) ultrasound machine. Prior to treatment and one year after SVR12, each patient underwent an ultrasonographic examination of the liver, gall bladder, spleen, and kidneys. In order to assess the severity of liver steatosis, we used the Hamaguchi Score (HS) [14], which evaluates hepatorenal echo contrast, liver brightness, deep attenuation of the echoes and vessel blurring. Portal vein Doppler examination and splenic measurement were available only one year after SVR12. Their acquisition methods were described elsewhere [15,16,17]. In addition, each patient underwent LSM (prior to treatment and one year after SVR12) and spleen stiffness measurement (SSM) (one year after SVR12), with point shear wave elastography (pSWE) using the ElastPQ evaluation protocol. Their acquisition methods and reliability characteristics are described elsewhere [18]. We used the following cut-off values: mild/no fibrosis (F0-1), 4.1–5.5 kPa; significant fibrosis (F2), 5.51–7.5 kPa; advanced fibrosis (F3), 7.51–11.00 kPa; liver cirrhosis (F4) > 11.01 kPa [19,20].

### 2.2. Inclusion and Exclusion Criteria

Inclusion criteria were: (1) age >18 years, (2) HCV infection confirmed by positive HCV-RNA titers, (3) intention to treat with DAAs, (4) significant liver fibrosis as demonstrated by pSWE (F ≥ 3–4 according to METAVIR staging, LSM ≥ 7.51) [21]. We also excluded patients with active hepatitis B virus (HBV), human immunodeficiency virus (HIV) infection, and hepatitis A and hepatitis E viruses (HAV/HEV).

We excluded patients with HS >3 and serum cholesterol >250 mg/dL. We also excluded patients with factors that could potentially influence splenoportal dynamics, including pregnant women, patients with current alcohol abuse, decompensating events (such as hepatic encephalopathy, variceal hemorrhage, ascites, and spontaneous bacterial peritonitis), previous endoscopic EVs banding ligation, ongoing intake of nonselective beta-blockers (NSBB), history of portal vein thrombosis, placement of transjugular intrahepatic portosystemic shunt (TIPS), noncirrhotic causes of PH, current/recent diagnosis of hepatocellular carcinoma [22], presence of collateral hepatofugal shuntings and signs of liver failure. We also excluded patients with heart failure and/or documented congestive hepatopathy.

### 2.3. Statistical Analysis

According to the size of our sample, the Shapiro–Wilk test was performed to verify the normal distribution of variables. Normally distributed variables were reported as mean (±standard deviation, SD), whereas other variables were reported as median (Quartile 1;Quartile 3). Differences between continuous variables were examined using the Student *t*-test (if normally distributed) or Mann–Whitney U test (if not-normally distributed). Patients were divided into four groups according to fibrosis downstaging: group 1 (G1), consisted of patients who had LSM compatible with F4 one year after SVR12; group 2 (G2), consisted of patients who had LSM compatible with F4 prior to treatment and F3 one year after SVR12; group 3 (G3), consisted of patients who had LSM compatible with F ≥3 and F2 one year after SVR12; group 4 (G4), consisted of patients who had LSM compatible with F ≥3 and F0-1 one year after SVR12. We did not employ any corrections for multiple comparisons.

To determine the collinearity between variables, we employed the variance inflation factor (VIF). Liver fibrosis overestimation probability was studied through logistic regression models [23]. In particular, the risk of overestimation of one stage of fibrosis (M1), was calculated using as dependent variable 0 = no (i.e., patient did not experience downstage of one fibrosis stage) and 1 = yes (patient experienced downstage of one fibrosis stage), and serum AST and ALT as independent variables. The risk of overestimation of two or more stages of fibrosis (M2), was also calculated using as dependent variable 0 = no (i.e., patient did not experience downstage of two or more fibrosis stages) and 1 = yes (patient experienced downstage of two or more fibrosis stages), and as independent variable serum AST and ALT. The independent variables were all modeled as continuous. In order to calculate the expected probability plot, each linear predictor (*LP*) was employed in the following function:

(1) $$f\left(LP\right)=1-\left(\frac{1}{1+\;e^{LP}}\right)$$

We compared models using the Akaike information criterion (AIC) and the Bayesian information criterion (BIC) and calculated Nagelkerke pseudo-R^2^ and the area under the receiver-operating characteristic curve (AUROC). For all analyses, two-sided statistical significance was defined as *p* < 0.05. Data were analyzed using SPSS (statistical package for social science) version 25.0 (IBM SPSS Statistics for MAC OS. Armonk, NY: IBM Corp.)

## 3. Results

From an original cohort of 133 patients who met the inclusion criteria, 12 were lost to follow-up, 10 had unreliable LSM, and one was excluded for treatment failure. Therefore, 110 patients were included in the study, of which 48 (43.6%) were males, with a median age of 64 (52;74) years. Sixty-three patients (57.2%) had genotype 1 infection, 14 (12.7%) genotype 2, 25 (22.7%) genotype 3 and 8 (7.3%) genotype 4. Median viral load prior to therapy was 1.81 (1.10;2.23) million IU/mL, with 34% patients having an undetectable viral load by four weeks, and 97% of patients with an undetectable viral load by eight weeks, and 108 (98.1%) by the twelfth week. Variations of laboratory values from baseline are reported in Table 1: a statistically significant decrease was detected in serum AST (*p* < 0.001), ALT (*p* < 0.001), and platelet count (*p* < 0.001). Median baseline LSM was 14.10 (11.3;20) kPa, which decreased to 7.1 (5.3;11) kPa 1-year after treatment (*p* < 0.001). In particular, 60 (55%) patients had baseline LSM compatible with F4 stage, while 50 (45%) with F3 stage. One year after conclusion of therapy, 34 (30.9%) patients had LSM compatible with F4 stage, 9 (8.1%) with F3 stage, 30 (27.2%) with F2 stage, and 37 (33.63%) with F0-1 stages. Accordingly, patients were stratified by degree of fibrosis decrease, and inter-groups median values of AST, ALT, and platelets were compared, as shown in Table 2. Patients who maintained LSM compatible with F4 fibrosis staging had lower values of AST (62 vs. 176 IU/L, *p* < 0.001), ALT (71 vs. 251 IU/L, *p* < 0.001), and platelet counts (97 vs. 222 × 10^9^/L, *p* < 0.001) if compared to patients who experienced LSM regression to values compatible with F0-1 stages. In addition, (as shown in Table 3) median spleen stiffness values were 45 (36;56) kPa in patients who maintained LSM values compatible with F4 stage, which were higher if compared to patients who showed lower degree of fibrosis: F3 (29 kPa, *p* = 0.001), F2 (24 kPa, *p* = 0.001) and F0-1 (19 kPa, *p* < 0.001). In addition, patients who maintained LSM compatible with F4 showed higher spleen area if compared to all other groups (Table 3). In particular, the difference in median SSM (45 vs. 19 kPa, *p* < 0.001) and spleen area (54 vs. 38 cm^2^, *p* < 0.001) were more marked between group 1 and group 4. Regarding portal vein flow velocity, patients in group 1 had slower values if compared to other groups, excluding group 1 and group 2 comparison. Inter-group differences of portal vein diameter were not statistically significant, except for group 1 and group 4 (1.14 vs. 1.01 cm, *p* = 0.043).

### Models of Fibrosis Overestimation

**Figure 1:**
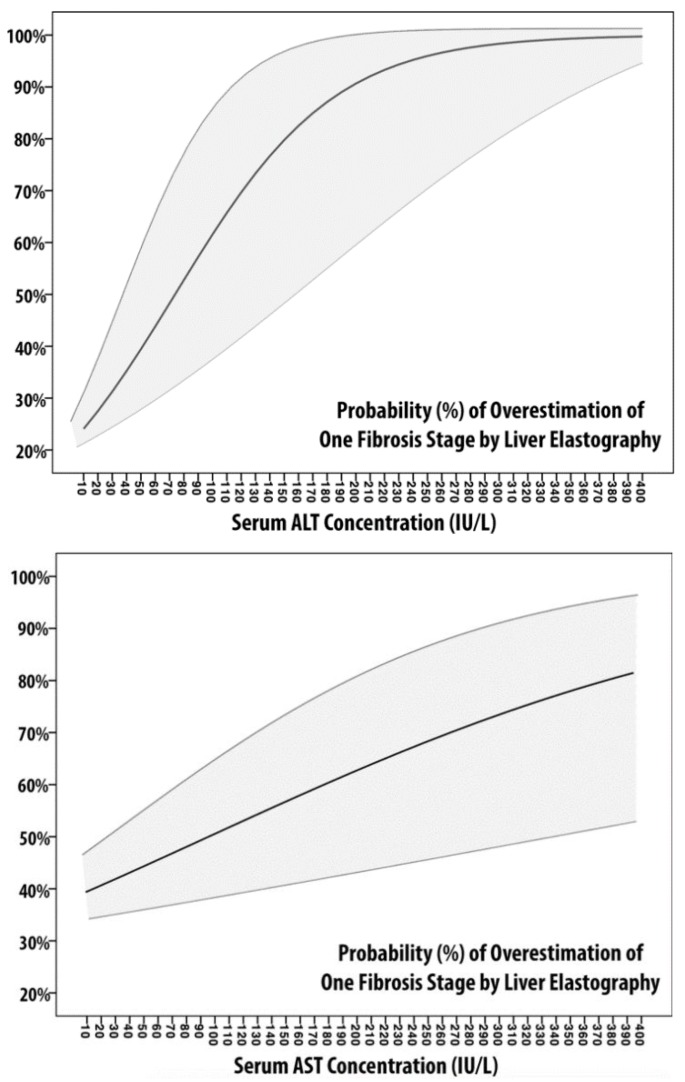
Probability Plot of Models 1. The two figures represent the probability of overestimation of one fibrosis stage by liver elastography measurement according to serum ALT (left) and serum AST (right). Logistic regression coefficients of probability are represented with 95% confidence intervals (light gray area).

We built four models according to logistic regression analysis, whose discriminative and calibration metrics are described in Table 4. In M1-AST and M1-ALT, the dependent variable is represented by the overestimation of one grade of fibrosis, whereas the independent variables are AST and ALT, respectively. The probability of overestimation of one grade of fibrosis is plotted in Figure 1. The two probability plots present different graphical behaviors: while the M1-ALT shape could be approximated to that of an S-shaped curve, M1-AST is almost linear. According to Table 4, M1-ALT showed better calibration (lowest AIC/BIC and highest Pseudo-R2) but equal discriminative ability. Also, according to these models, there is a probability to overestimate one grade of fibrosis >75% for ALT ≥ 150 IU/L (3.3 ULN) and AST ≥ 335 IU/L (7.4 ULN).

**Figure 2:**
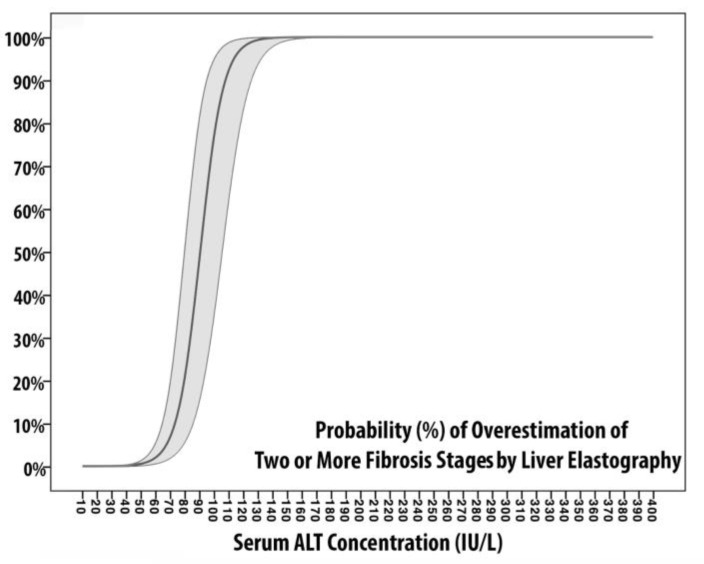

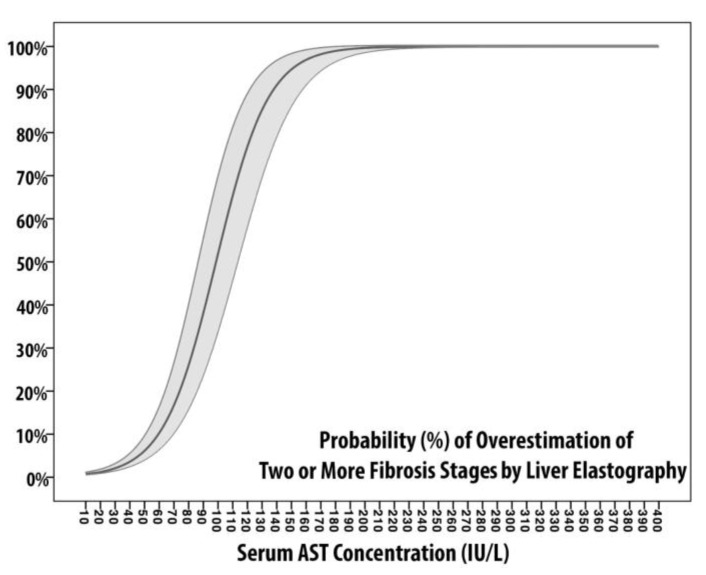
Probability plot of Models 2. The two figures represent the probability of overestimation of two or more fibrosis stages by liver elastography measurement according to serum ALT (left) and serum AST (right). Logistic regression coefficients of probability are represented with 95% confidence intervals (light gray area).

In M2-AST and M2-ALT, the dependent variable is represented by the overestimation of two or more grades of fibrosis, whereas the independent variables are AST and ALT, respectively. The probability of overestimation of two or more grades of fibrosis is plotted in Figure 2. Although both curves are S-shaped, the M2-ALT slope increases more rapidly; from 0% at 50 IU/L to 99% at 127 IU/L. The two curves intersect at 84 IU/L, where the probability is equal to 46%. Also, M2-ALT reaches the superior plateau (equal to 100% of probability) at 140 IU/L, whereas M2-AST approaches it at 211 IU/L. In summary, the probability of overestimation of two fibrosis grade is equal to (1) 50% for AST of 99 IU/L (2.2 ULN) and ALT of 90.5 IU/L (2 ULN), (2) 80% for AST of 123.5 IU/L (2.7 ULN) and ALT of 101.5 IU/L (2.25 ULN), and (3) reaches the superior plateau for AST of 211 IU/L (4.7 ULN) and ALT of 140 IU/L (3.1 ULN). According to Table 4, M2-ALT showed slightly better calibration (AIC = 88, BIC = 90, Pseudo-R2 = 0.785) and discriminative ability (AUROC = 0.958) if compared to M2-AST (AIC = 89, BIC = 92, Pseudo-R2 = 0.682, AUROC = 0.936). However, both M2s are superiorly calibrated, more discriminant, and were built on variables with a lesser degree of collinearity (VIF equivalent to 1.712 and 2.532, respectively) if compared to M1-models.

## 4. Discussion

It is commonly assumed that aminotransferase elevation is mainly related to liver cellular damage when hepatocytes undergo necrosis as a result of direct cellular damage or inflammation [24,25,26]. In the case of HCV, the cytolysis of infected hepatocytes is mediated by perforin and granzyme B produced by cytotoxic T-lymphocytes [27]. Also, serum transaminase concentrations (mostly ALT) have been linked to the histological grading of necroinflammatory activity, and, as expected, patients with elevated ALT generally show higher activity scores [28,29]. These concepts should be taken into consideration for a critical evaluation of liver elastography results as it has been proven that stiffness values are influenced by tissue congestion and inflammation, rather than the mere degree of collagen deposition (i.e., fibrosis) [30,31]. Therefore, it is safe to assume, as, in the case of our study, that hepatic congestion and inflammation can affect LSM, and that serum transaminase levels can be used to quantify the amount of ongoing damage. In this regard, even if their utility is still in debate [32], inflammation-adapted LSM cut-offs have been proposed by Mueller et al. [33]. In addition, successful viral eradication has been associated with a decline in LSM. According to a recent meta-analysis which included 24 studies, six to twelve months after achieving SVR12, patients experience a median LSM decrease of 28%, and approximately 47% of patients with baseline values compatible with advanced liver fibrosis or cirrhosis show post-treatment values compatible with lower degrees of fibrosis [34]. Also, in light of recent findings, the decrease of LSM appears to be more evident in patients with baseline ALT ≥ 2ULN [35]. In our cohort of patients, LSM showed a median decrease of 35% from baseline values, and 67 (61%) patients showed post-treatment values compatible with lower fibrosis stages. Notably, the patients who showed LSM compatible with F0-1 one year after SVR 12 (Table 2), were the ones with higher serum transaminases levels (median AST 3.9 ULN and median ALT 5.6 ULN), whereas those without fibrosis downstaging had lower median levels of transaminases (median AST 1.4 ULN and median ALT 1.6 ULN). These observations may suggest that the principal driver of LSM improvements is related to the suppression of virus-induced liver inflammation, as a consequence of successful viral eradication rather than a simple regression of liver fibrosis. This hypothesis is what drove us in trying to quantify the degree of overestimation related to serum transaminases: we developed M1s and M2s using as dependent variables the downstaging of one fibrosis stage (M1s) or two or more fibrosis stages (M2s) and serum transaminases as independent variables. It should be taken into account that the logistic regression models were built on the assumption that one year is a relatively short period for histological improvement of liver fibrosis, especially in patients who are supposed to have significant liver fibrosis [36,37,38]. Regarding Table 4, both M1s have significantly less performant calibration and discriminative metrics if compared to M2s. The slope of M2-ALT increases more rapidly from 50 IU/L to 127 IU/L, which means that variations in the probability of overestimation for the same unitary increment are peculiar to each transaminase. Also, M2-ALT showed slightly better calibration (lowest AIC/BIC and highest Pseudo-R2) and discriminative ability (highest AUROC) if compared to M2-AST. According to Figure 2, the probability of overestimation of two fibrosis grade is equal to 50% for serum transaminases around 2–2.2 ULN and steadily increase to 100% for serum transaminases between 3.1 and 4.7 ULN.

Alternatively, according to the methods section, these results can be interpreted as follows: what is the probability of LSM downstaging (≥2 fibrosis stage) at one year of follow-up, starting from values compatible with severe liver fibrosis, and considering baseline serum transaminase? As stated above, it is very unlike to obtain real downstaging in such a short follow-up period. However, it would be appropriate to detect accurate LSM after the elimination of such a robust confounding factor as inflammation. Other parameters can be used for appropriate liver fibrosis staging and, in particular, ultrasonographic signs of severe liver fibrosis. For example, SSM has been found to correlate with liver fibrosis staging [39,40]; besides, spleen size and platelet count are often used as noninvasive indicators of liver cirrhosis [41,42]. Prior to therapy, patients in G1 had significantly lower platelet counts if compared to all other groups (Table 2). In addition, one year after SVR12, each fibrosis group was characterized by different ranges of SSM and spleen areas (Table 3). Although it could be challenging to differentiate between each fibrosis stage, it is possible to use SSM values to rule-in liver cirrhosis: G1 showed statistically higher SSM if compared to other groups.

The most important limitation of this study is the absence of the histological assessment of liver fibrosis, both at baseline evaluation and during follow-up, which is a common problem in the majority of the studies that are addressing this issue in the DAAs era. However, the real novelty of this study is related to the quantification of the influence of elevated serum transaminase levels in LSM, and the creation of a probability model that can be easily read and employed in the everyday clinical practice. We believe that our study may serve as a reference point for appropriate fibrosis stratification by liver elastography in patients with active HCV infection.

## Figures and Tables

**Table 1:** Patients’ laboratory values prior to treatment, end of treatment, and one year after SVR 12. ^†^ represents a *p* < 0.001, expressed as a comparison between baseline and one-year after SVR12; ^‡^ represents a *p* < 0.001 expressed as a comparison between end of treatment and one-year after SVR12. AST: aspartate aminotransferase; ALT: alanine aminotransferase; GGT: gamma-glutamyl transpeptidase; ALP: alkaline phosphatase.

Values	Baseline	End of Therapy	One-Year after SVR12
Male, *n* (%)	48 (43.6%)		
Age, years	64 (52;74)		
HCV-Genotype, *n* (%)			
Genotype 1	63 (57.2%)		
Genotype 2	14 (12.7%)		
Genotype 3	25 (22.7%)		
Genotype 4	8 (7.3%)		
BMI, (kg/m^2^)	21.1 (17.7; 23.4)	22 (18;24)	23.2 (20.2;25.6)
Creatinine, (mg/dL)	0.78 (0.70;0.84)	0.81 (0.73;0.91)	0.76 (0.69;0.82)
Glucose, (mg/dL)	94 (85;102) ^†^	98 (86;102) ^‡^	105 (94;113) ^†,^^‡^
Total Cholesterol, (mg/dL)	180 (170;200) ^†^	181 (176;210) ^‡^	193 (180;223) ^†,^^‡^
AST, (IU/L)	90 (68;149) ^†^	35 (31;42) ^‡^	21.5 (20;26) ^†,^^‡^
ALT, (IU/L)	94.5 (79;135) ^†^	32 (28;36) ^‡^	17.5 (15;21) ^†,^^‡^
GGT, (IU/L)	44 (37;46)	43 (35;48)	40 (32;50)
ALP (IU/L)	105 (80;130)	100 (78;135)	107 (89;116)
Total Bilirubin, (mg/dL)	0.85 (0.75;0.99)	0.80 (0.68;1.12)	0.87 (0.70;1.01)
Albumin, (g/dL)	4.1 (3.5;4.4)	3.8 (3.4;4.3)	3.9 (3.6;4.5)
INR	1.10 (1.03;1.14)	1.07 (1.04;1.09)	1.12 (1.07;1.15)
PLT, (×10^3^/L)	151 (137.5;218) ^†^	165 (153;171) ^‡^	185 (152.5;242) ^†,^^‡^
Liver Stiffness, (kPa)	14.10 (11.3;20) ^†^		7.1 (5.3;11) ^†^

**Table 2:** Patients were stratified into four groups based on liver fibrosis downstaging according to liver stiffness values. Values were reported according to their Median and Quartile 1–Quartile 3, Me (Q1;Q3). Inter-group differences were evaluated using Mann–Whitney U test. AST: aspartate aminotransferase; ALT: alanine aminotransferase; NS: not statistically significant difference; NE: not evaluated.

Patients Stratified by Degree of Fibrosis Regression	AST (IU/L) (Baseline)	ALT (IU/L) (Baseline)	Platelets (×10^9^/L) (Baseline)	AST (IU/L) (1-Year after Therapy)	ALT (IU/L) (1-Year after Therapy)	Platelets (×10^9^/L) (1-Year after Therapy)	% Changes in LSM from Baseline
**Group 1 (G1)** (*n* = 34) *From F4 to F4*	62 (28.5;85)	71 (41.5;87)	97 (80;130)	26 (20;34)	18 (14;29.5)	115 (90;153)	−14.3%
**Group 2 (G2)** (*n* = 9) *From F4 to F3*	70 (51;90)	82 (52;118)	152 (140;217)	21 (19;26.5)	15 (13;22.5)	188 (151;239)	−20%
**Group 3 (G3)** (*n* = 30) *From F3/4 to F2*	110 (90;150)	100 (89;119)	170 (150;240)	20 (18;24)	16 (15;21)	190 (170;259)	−37%
**Group 4 (G4)** (*n* = 37) *From F3/4 to F0-1*	176 (149;230)	251 (182;300)	222 (146;291)	22 (20;24)	21 (17;26)	251 (182;300)	−57.5%
**Statistical Significance**							
Group 1 vs. Group 2	*p* = 0.049	*p* = 0.043	*p* = 0.019	NS	NS	*p* = 0.022	NE
Group 1 vs. Group 3	*p* = 0.03	*p* = 0.001	*p* = 0.001	NS	NS	*p* < 0.001	NE
Group 1 vs. Group 4	*p* < 0.001	*p* < 0.001	*p* < 0.001	NS	NS	*p* < 0.001	NE
Group 2 vs. Group 3	*p* = 0.032	NS	*p* = 0.047	NS	NS	NS	NE
Group 3 vs. Group 4	*p* = 0.028	*p* = 0.021	*p* = 0.032	NS	NS	*p* = 0.039	NE

**Table 3:** Patients were stratified into four groups based on liver fibrosis downstaging according to liver stiffness values. Values were reported according to their Median and Quartile 1–Quartile 3, Me (Q1;Q3). Inter-group differences were evaluated using Mann–Whitney U test. NS: not statistically significant difference.

Patients Stratified by Degree of Fibrosis Regression	Spleen Stiffness (kPa)	Portal Vein Diameter (cm)	Portal Vein Median Flow Velocity (cm/s)	Spleen Bipolar Diameter (cm)	Spleen Area (cm^2^)
**Group 1 (G1)** (*n* = 34) *From F4 to F4*	45 (36;56)	1.14 (1;1.3)	17.8 (17.5;18)	12.4 (11;13.6)	54 (46.5;68)
**Group 2 (G2)** (*n* = 9) *From F4 to F3*	29 (27;32)	1.01 (0.9;1.18)	19 (15;21)	11.5 (10.5;13.5)	48 (38;66)
**Group 3 (G3)** (*n* = 30) *From F3/4 to F2*	24 (18;26)	1.01 (0.9;1.06)	20.4 (18.2;24)	10.7 (10;12)	44 (35;56)
**Group 4 (G4)** (*n* = 37) *From F3/4 to F0-1*	19 (16;24)	1.01 (0.9;1.09)	26 (17;28)	10 (9;11)	38 (30;44.5)
**Statistical Significance**					
Group 1 vs. Group 2	*p* = 0.01	NS	NS	NS	*p* = 0.039
Group 1 vs. Group 3	*p* = 0.001	NS	*p* = 0.024	*p* = 0.05	*p* = 0.023
Group 1 vs. Group 4	*p* < 0.001	*p* = 0.043	*p* = 0.001	*p* = 0.001	*p* < 0.001
Group 2 vs. Group 3	*p* = 0.038	NS	*p* = 0.045	NS	*p* = 0.037
Group 3 vs. Group 4	*p* = 0.029	NS	*p* = 0.031	*p* = 0.044	*p* = 0.036

**Table 4:** Association between serum transaminase and liver fibrosis overestimation. Models were realized according to fibrosis measurement one-year after SVR12. VIF: variance inflation factor; AIC: Aikake information criterion; BIC: Bayesian information criterion; AUROC: area under the receiver-operating characteristic curve.

	Model 1 (M1) (Overestimation of One Fibrosis Stage)		Model 2 (M2) (Overestimation of Two or More Fibrosis Stages)	
**Model Parameters**	**M1-AST**	**M1-ALT**	**M2-AST**	**M2-ALT**
**Linear Predictor**	−0.479 + 0.005×[AST]	−1.329 + 0.018×[ALT]	−5.525 + 0.056×[AST]	−11.497 + 0.127×[ALT]
**VIF**	2.532		1.712	
**AIC**	210	209	89	88
**BIC**	213	200	92	90
**AUROC**	0.623 (0.4;0.685)	0.623 (0.485;0.761)	0.936 (0.882;0.989)	0.958 (0.922;0.996)
**Nagelkerke-PseudoR2**	0.009	0.088	0.682	0.785
***Hosmer-Lemeshow p-value***	0.048	0.025	0.203	0.96

## Abbreviations

HCV	hepatitis C virus
DAAs	direct antiviral agents
NITs	noninvasive techniques
ALT	alanine aminotransferase
AST	aspartate aminotransferase
ULN	upper limit of normality
LSM	liver stiffness measurement
SSM	spleen stiffness measurement
SVR	sustained viral response
pSWE	point-shear wave elastography

## References

[1] Blach S., Zeuzem S., Manns M., Altraif I., Duberg A.S., Muljono D.H., Waked I., Alavian S.M., Lee M.H., Negro F. (2017). Global prevalence and genotype distribution of hepatitis C virus infection in 2015: A modelling study. Lancet Gastroenterol. Hepatol..

[2] Lingala S., Ghany M.G. (2015). Natural History of Hepatitis C. Gastroenterol. Clin. North Am..

[3] Chen Q., Ayer T., Bethea E., Kanwal F., Wang X., Roberts M., Zhuo Y., Fagiuoli S., Petersen J., Chhatwal J. (2019). Changes in hepatitis C burden and treatment trends in Europe during the era of direct-acting antivirals: A modelling study. BMJ Open.

[4] Pawlotsky J.M., Negro F., Aghemo A., Berenguer M., Dalgard O., Dusheiko G., Marra F., Puoti M., Wedemeyer H. (2018). EASL Recommendations on Treatment of Hepatitis C 2018. J. Hepatol..

[5] Barr R.G., Ferraioli G., Palmeri M.L., Goodman Z.D., Garcia-Tsao G., Rubin J., Garra B., Myers R.P., Wilson S.R., Rubens D. (2016). Elastography Assessment of Liver Fibrosis: Society of Radiologists in Ultrasound Consensus Conference Statement. Ultrasound Q..

[6] Sigrist R.M.S., Liau J., El Kaffas A., Chammas M.C., Willmann J.K. (2017). Ultrasound elastography: Review of techniques and clinical applications. Theranostics.

[7] Arena U., Vizzutti F., Corti G., Ambu S., Stasi C., Bresci S., Moscarella S., Boddi V., Petrarca A., Laffi G. (2008). Acute viral hepatitis increases liver stiffness values measured by transient elastography. Hepatology.

[8] Li Q., Chen L., Zhou Y. (2018). Diagnostic accuracy of liver stiffness measurement in chronic hepatitis B patients with normal or mildly elevated alanine transaminase levels. Sci. Rep..

[9] Fung J., Lai C.L., Chan S.C., But D., Seto W.K., Cheng C., Wong D.K.H., Lo C.M., Fan S.T., Yuen M.F. (2010). Correlation of liver stiffness and histological features in healthy persons and in patients with occult hepatitis B, chronic active hepatitis B, or hepatitis B cirrhosis. Am. J. Gastroenterol..

[10] Fung J., Lai C.L., Cheng C., Wu R., Wong D.K.H., Yuen M.F. (2011). Mild-to-moderate elevation of alanine aminotransferase increases liver stiffness measurement by transient elastography in patients with chronic hepatitis B. Am. J. Gastroenterol..

[11] Vispo E., Barreiro P., del Valle J., Maida I., de Ledinghen V., Quereda C., Moreno A., Macías J., Castera L., Pineda J.A. (2009). Overestimation of liver fibrosis staging using transient elastography in patients with chronic hepatitis C and significant liver inflammation. Antivir. Ther..

[12] Masutti F., Giuffrè M., Santi M., Crosato M.I., Roberto L., Balestra R., Crocè L.S. (2019). How does Trieste treat HCV in PWID? An effective coordination between addiction treatment service (SerD), the infective disease department and the liver clinic. Dig. Liver Dis..

[13] Martinot-Peignoux M., Stern C., Maylin S., Ripault M.P., Boyer N., Leclere L., Castelnau C., Giuily N., Ray A.E., Cardoso A.C. (2010). Twelve weeks posttreatment follow-up is as relevant as 24 weeks to determine the sustained virologic response in patients with hepatitis c virus receiving pegylated interferon and ribavirin. Hepatology.

[14] Hamaguchi M., Kojima T., Itoh Y., Harano Y., Fujii K., Nakajima T., Kato T., Takeda N., Okuda J., Ida K. (2007). The Severity of Ultrasonographic Findings in Nonalcoholic Fatty Liver Disease Reflects the Metabolic Syndrome and Visceral Fat Accumulation. Am. J. Gastroenterol..

[15] Giuffrè M., Macor D., Masutti F., Abazia C., Tinè F., Patti R., Buonocore M.R., Colombo A., Visintin A., Campigotto M. (2019). Can we revise Baveno VI criteria using spleen elastography?. Dig. Liver Dis..

[16] Macor D., Giuffrè M., Masutti F., Abazia C., Tinè F., Patti R., Buonocore M.R., Colombo A., Visintin A., Campigotto M. (2019). Spleen and liver elastography as a non-invasive tool for detection of esophageal varices in patients with liver cirrhosis. Dig. Liver Dis..

[17] Giuffrè M., Macor D., Masutti F., Abazia C., Tinè F., Patti R., Buonocore M.R., Colombo A., Visintin A., Campigotto M. (2018). The Role of Liver and Spleen Elastography in the Screening of Esophageal Varices in Patients with Liver Cirrhosis: Do We Really Need Endoscopy?. Hepatology.

[18] Giuffrè M., Macor D., Masutti F., Tinè F., Bedogni G., Tiribelli C., Crocè L.S. (2019). Spleen Stiffness Probability Index (SSPI): A Simple and Accurate Method to Detect Esophageal Varices in Patients with Compensated Liver Cirrhosis. Ann. Hepatol..

[19] Ferraioli G., Maiocchi L., Lissandrin R., Tinelli C., De Silvestri A., Filice C., Above E., Barbarini G., Bruno R., Corona S. (2016). Accuracy of the ElastPQ ^®^ technique for the assessment of liver fibrosis in patients with chronic hepatitis C: A “real life” single center study. J. Gastrointest. Liver Dis..

[20] Giuffrè M., Macor D., Masutti F., Abazia C., Tinè F., Patti R., Buonocore M.R., Colombo A., Visintin A., Campigotto M. (2019). Evaluation of spleen stiffness in healthy volunteers using point shear wave elastography. Ann. Hepatol..

[21] Ferraioli G., Tinelli C., Lissandrin R., Zicchetti M., Bello B.D., Filice G., Filice C. (2014). Point shear wave elastography method for assessing liver stiffness. World J. Gastroenterol..

[22] Giuffrè M., Patti R., Pascut D., Sukowati C., Masutti F., Abazia C., Tinè F., Macor D., Buonocore M.R., Colombo A. (2018). MicroRNAs as Regulators of Neo-Angiogenesis in Hepatocellular Carcinoma. Ann. Gastroenterol. Dig. Disord..

[23] Fagerland M.W., Hosmer D.W. (2013). A goodness-of-fit test for the proportional odds regression model. Stat. Med..

[24] Leist M., Gantner F., Bohlinger I., Tiegs G., Germann P.G., Wendel A. (1995). Tumor necrosis factor-induced hepatocyte apoptosis precedes liver failure in experimental murine shock models. Am. J. Pathol..

[25] Lawson J.A., Fisher M.A., Simmons C.A., Farhood A., Jaeschke H. (1998). Parenchymal cell apoptosis as a signal for sinusoidal sequestration and transendothelial migration of neutrophils in murine models of endotoxin and Fas-antibody-induced liver injury. Hepatology.

[26] Bajt M.L. (2000). Protection against Fas Receptor-Mediated Apoptosis in Hepatocytes and Nonparenchymal Cells by a Caspase-8 Inhibitor in Vivo: Evidence for a Postmitochondrial Processing of Caspase-8. Toxicol. Sci..

[27] Fitzmaurice K., Klenerman P. (2008). Cellular Immunity and Acute Hepatitis C Infection. Curr. Pharm. Des..

[28] Shen F.F., Wang Y., Wang Y.F., Zheng R.D., Xian J.C., Shi J.P., Qu Y., Dong Y.W., Xu M.Y., Lu L.G. (2018). Prediction of hepatic necroinflammatory activity in patients with chronic hepatitis B by a simple noninvasive model. J. Transl. Med..

[29] Pradat P., Alberti A., Poynard T., Esteban J.I., Weiland O., Marcellin P., Badalamenti S., Trépo C. (2002). Predictive value of ALT levels for histologic findings in chronic hepatitis C: A European collaborative study. Hepatology.

[30] Xie L.T., Xu D.X., Tian G., Zhong L.Y., Zhao Q.Y., Ke Q.H., Jiang T.A. (2018). Value of Two-Dimensional Shear Wave Elastography for Assessing Acute Liver Congestion in a Bama Mini-Pig Model. Dig. Dis. Sci..

[31] Piecha F., Paech D., Sollors J., Seitz H.K., Rössle M., Rausch V., Mueller S. (2018). Rapid change of liver stiffness after variceal ligation and TIPS implantation. Am. J. Physiol. Gastrointest. Liver Physiol..

[32] Castera L. (2015). Is it really worth adapting liver stiffness cut-offs according to AST levels?. Liver Int..

[33] Mueller S., Englert S., Seitz H.K., Badea R.I., Erhardt A., Bozaari B., Beaugrand M., Lupşor-Platon M. (2015). Inflammation-adapted liver stiffness values for improved fibrosis staging in patients with hepatitis C virus and alcoholic liver disease. Liver Int..

[34] Singh S., Facciorusso A., Loomba R., Falck-Ytter Y.T. (2018). Magnitude and Kinetics of Decrease in Liver Stiffness After Antiviral Therapy in Patients With Chronic Hepatitis C: A Systematic Review and Meta-analysis. Clin. Gastroenterol. Hepatol..

[35] Pons M., Santos B., Simón-Talero M., Ventura-Cots M., Riveiro-Barciela M., Esteban R., Augustin S., Genescà J. (2017). Rapid liver and spleen stiffness improvement in compensated advanced chronic liver disease patients treated with oral antivirals. Ther. Adv. Gastroenterol..

[36] Cammà C., Di Bona D., Schepis F., Heathcote J., Zeuzem S., Pockros P.J., Marceliln P., Balart L., Alberti A., Craxì A. (2004). Effect of Peginterferon Alfa-2a on Liver Histology in Chronic Hepatitis C: A Meta-analysis of Individual Patient Data. Hepatology.

[37] Shiratori Y., Imazeki F., Moriyama M., Yano M., Arakawa Y., Yokosuka O., Kuroki T., Nishiguchi S., Sata M., Yamada G. (2000). Histologic improvement of fibrosis in patients with hepatitis C who have sustained response to interferon therapy. Ann. Intern. Med..

[38] Marcellin P., Boyer N., Gervais A., Martinot M., Pouteau M., Castelnau C., Kilani A., Areias J., Auperin A., Benhamou J.P. (1997). Long-term histologic improvement and loss of detectable intrahepatic HCV RNA in patients with chronic hepatitis C and sustained response to interferon-α therapy. Ann. Intern. Med..

[39] Rewisha E.A., Elsabaawy M.M., Alsebaey A., Elmazaly M.A., Tharwa E.S. (2016). Evaluation of the role of liver and splenic transient elastography in chronic hepatitis C related fibrosis. J. Liver Dis. Transpl..

[40] Leung V.Y., Shen J., Wong V.W., Abrigo J., Wong G.L., Chim A.M., Chu S.H., Chan A.W., Choi P.C., Ahuja A.T. (2013). Quantitative Elastography of Liver Fibrosis and Spleen Stiffness in Chronic Hepatitis B Carriers: Comparison of Shear-Wave Elastography and Transient Elastography with Liver Biopsy Correlation. Radiology.

[41] Lurie Y., Webb M., Cytter-Kuint R., Shteingart S., Lederkremer G.Z. (2015). Non-invasive diagnosis of liver fibrosis and cirrhosis. World J. Gastroenterol..

[42] Giuffrè M., Campigotto M., Colombo A., Visintin A., Buonocore M.R., Aversano A., Budel M., Tinè F., Masutti F., Abazia C. (2019). Spleen Stiffness/Platelets-Based Models Can Predict Presence of Esophageal Varices in Patients With Compensated Liver Cirrhosis. Dig. Liver Dis..

